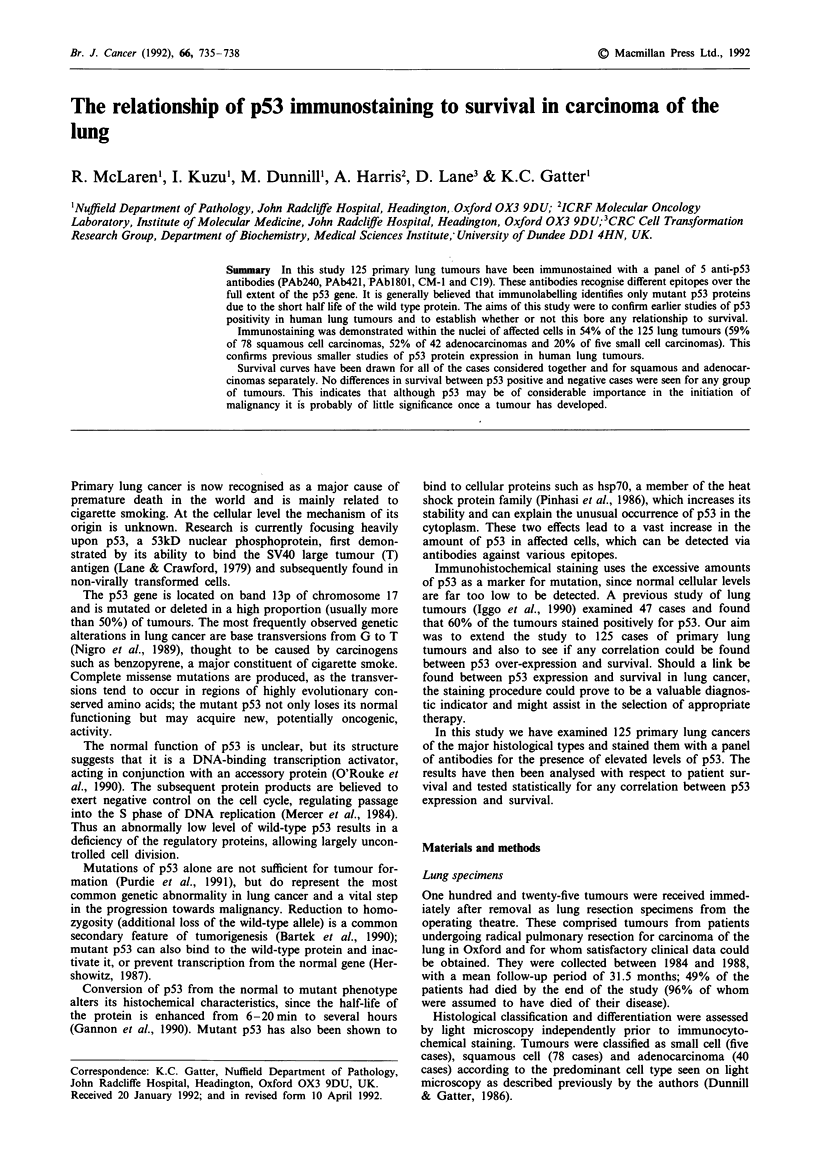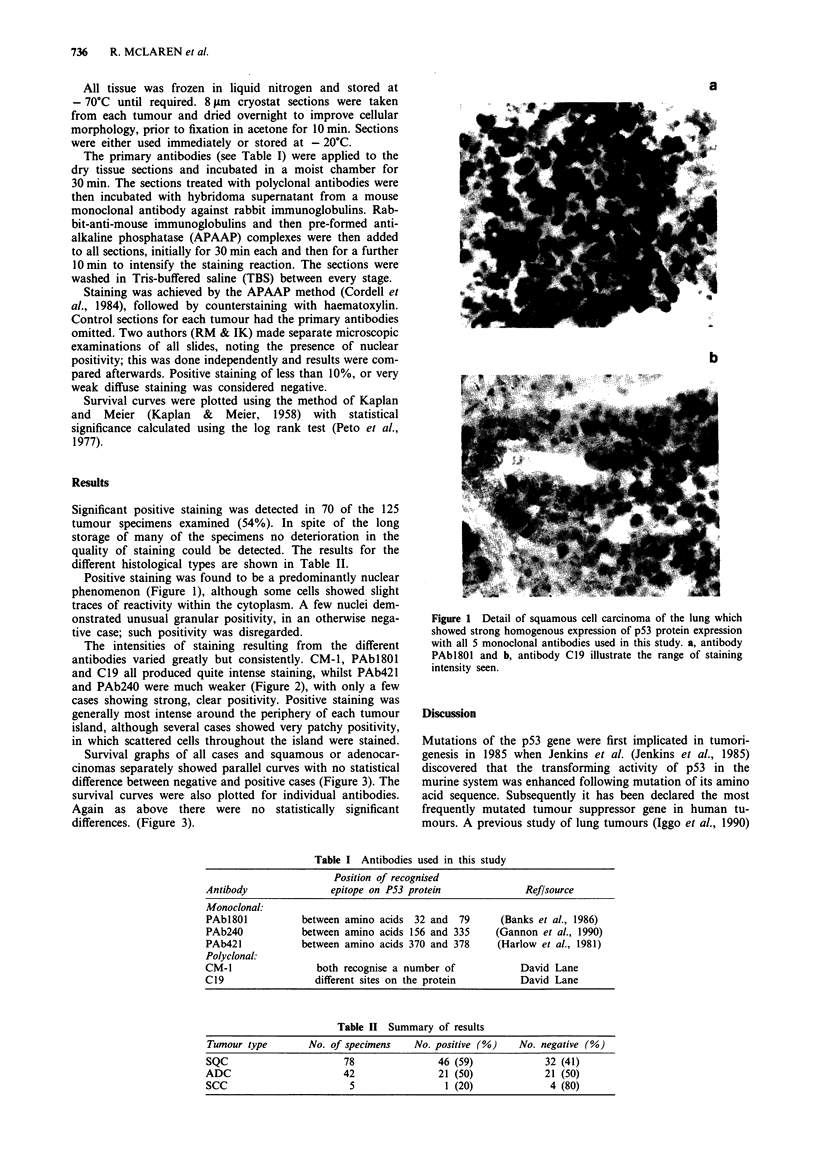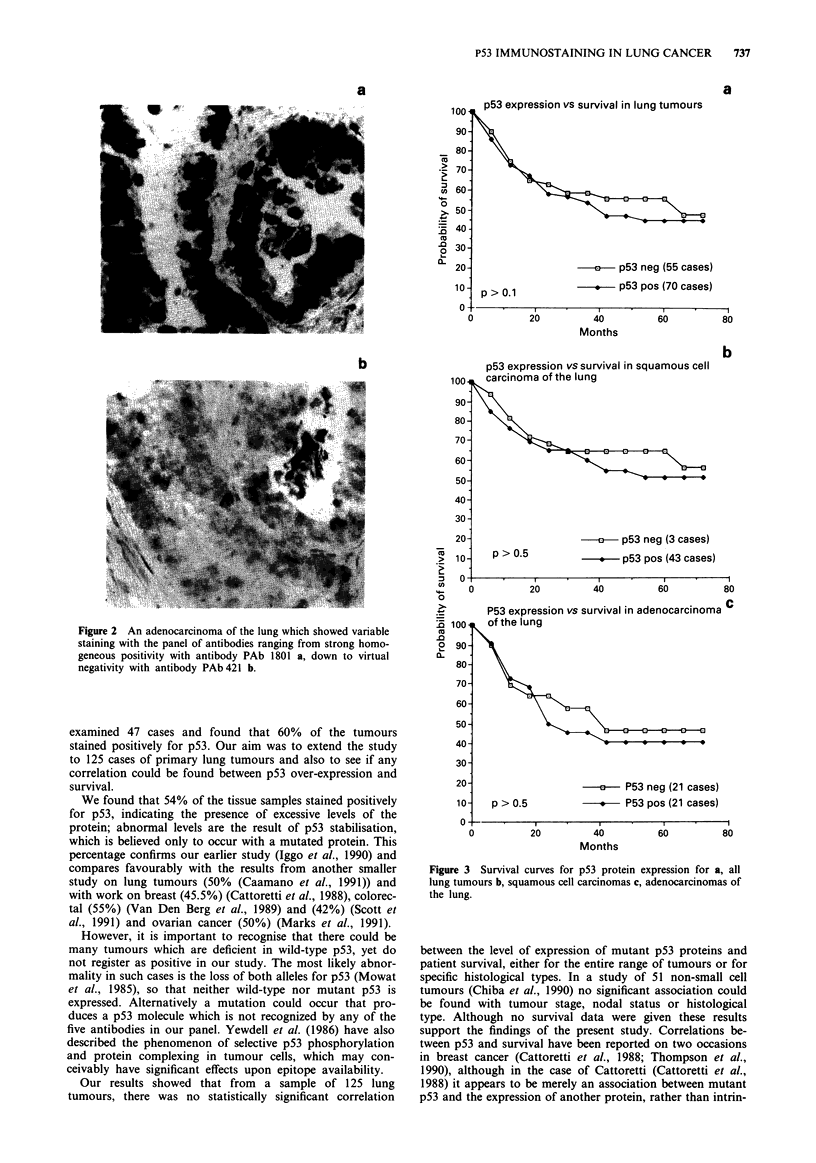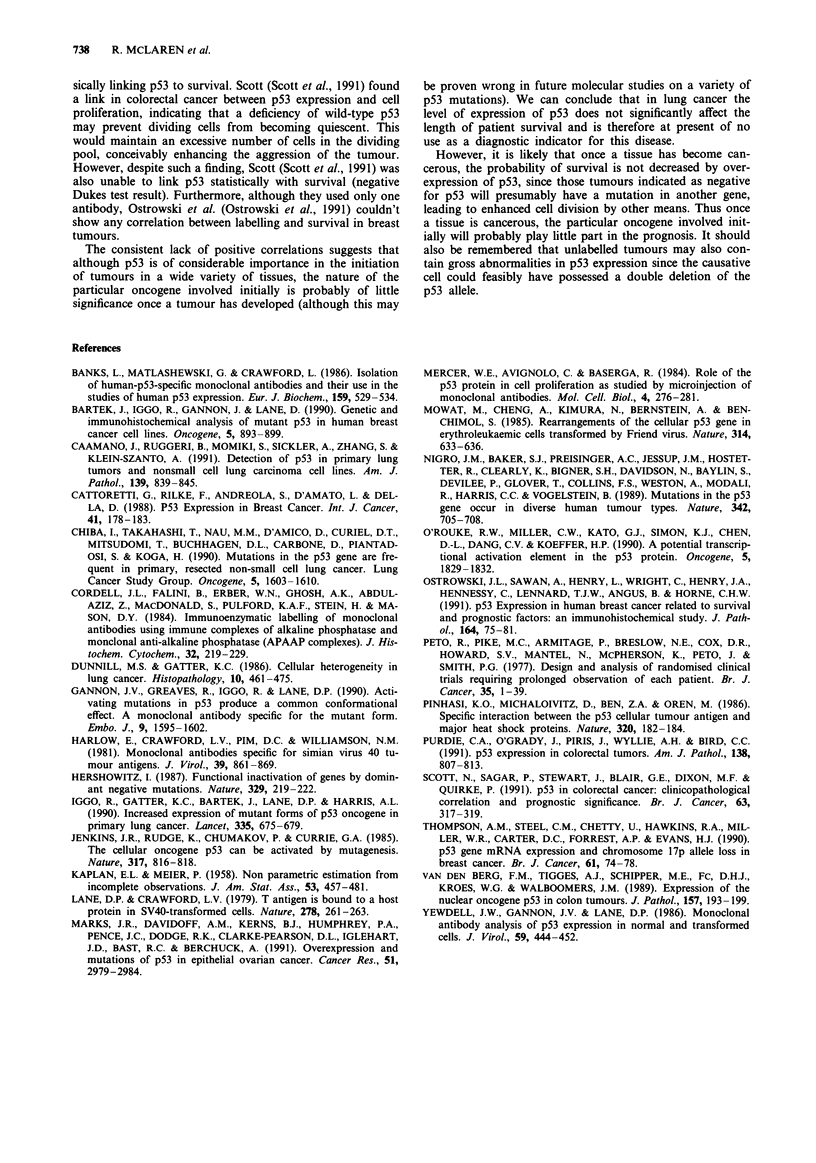# The relationship of p53 immunostaining to survival in carcinoma of the lung.

**DOI:** 10.1038/bjc.1992.348

**Published:** 1992-10

**Authors:** R. McLaren, I. Kuzu, M. Dunnill, A. Harris, D. Lane, K. C. Gatter

**Affiliations:** Nuffield Department of Pathology, John Radcliffe Hospital, Headington, Oxford, UK.

## Abstract

**Images:**


### The relationship of p53 immunostaining to survival in carcinoma of the lung

R. McLaren[1], I. Kuzu[1], M. Dunnill[1], A. Harris[2], D. Lane[3] & K.C. Gatter[1]

[1]*Nuffield Department of Pathology, John Radcliffe Hospital, Headington, Oxford OX3 9DU;* [2]*ICRF Molecular Oncology Laboratory, Institute of Molecular Medicine, John Radcliffe Hospital, Headington, Oxford OX3 9DU;* [3]*CRC Cell Transformation Research Group, Department of Biochemistry, Medical Sciences Institute, University of Dundee DD1 4HN, UK.*

**Summary** In this study 125 primary lung tumours have been immunostained with a panel of 5 anti-p53 antibodies (PAb240, PAb421, PAb1801, CM-1 and C19). These antibodies recognise different epitopes over the full extent of the p53 gene. It is generally believed that immunolabelling identifies only mutant p53 proteins due to the short half life of the wild type protein. The aims of this study were to confirm earlier studies of p53 positivity in human lung tumours and to establish whether or not this bore any relationship to survival.

Immunostaining was demonstrated within the nuclei of affected cells in 54% of the 125 lung tumours (59% of 78 squamous cell carcinomas, 52% of 42 adenocarcinomas and 20% of five small cell carcinomas). This confirms previous smaller studies of p53 protein expression in human lung tumours.

Survival curves have been drawn for all of the cases considered together and for squamous and adenocarcinomas separately. No differences in survival between p53 positive and negative cases were seen for any group of tumours. This indicates that although p53 may be of considerable importance in the initiation of malignancy it is probably of little significance once a tumour has developed.

Primary lung cancer is now recognised as a major cause of premature death in the world and is mainly related to cigarette smoking. At the cellular level the mechanism of its origin is unknown. Research is currently focusing heavily upon p53, a 53kD nuclear phosphoprotein, first demonstrated by its ability to bind the SV40 large tumour (T) antigen (Lane & Crawford, 1979) and subsequently found in non-virally transformed cells.

The p53 gene is located on band 13p of chromosome 17 and is mutated or deleted in a high proportion (usually more than 50%) of tumours. The most frequently observed genetic alterations in lung cancer are base transversions from G to T (Nigro *et al.*, 1989), thought to be caused by carcinogens such as benzopyrene, a major constituent of cigarette smoke. Complete missense mutations are produced, as the transversions tend to occur in regions of highly evolutionary conserved amino acids; the mutant p53 not only loses its normal functioning but may acquire new, potentially oncogenic, activity.

The normal function of p53 is unclear, but its structure suggests that it is a DNA-binding transcription activator, acting in conjunction with an accessory protein (O'Rouke *et al.*, 1990). The subsequent protein products are believed to exert negative control on the cell cycle, regulating passage into the S phase of DNA replication (Mercer *et al.*, 1984). Thus an abnormally low level of wild-type p53 results in a deficiency of the regulatory proteins, allowing largely uncontrolled cell division.

Mutations of p53 alone are not sufficient for tumour formation (Purdie *et al.*, 1991), but do represent the most common genetic abnormality in lung cancer and a vital step in the progression towards malignancy. Reduction to homozygosity (additional loss of the wild-type allele) is a common secondary feature of tumorigenesis (Bartek *et al.*, 1990); mutant p53 can also bind to the wild-type protein and inactivate it, or prevent transcription from the normal gene (Hershowitz, 1987).

Conversion of p53 from the normal to mutant phenotype alters its histochemical characteristics, since the half-life of the protein is enhanced from 6-20 min to several hours (Gannon *et al.*, 1990). Mutant p53 has also been shown to bind to cellular proteins such as hsp70, a member of the heat shock protein family (Pinhasi *et al.*, 1986), which increases its stability and can explain the unusual occurrence of p53 in the cytoplasm. These two effects lead to a vast increase in the amount of p53 in affected cells, which can be detected via antibodies against various epitopes.

Immunohistochemical staining uses the excessive amounts of p53 as a marker for mutation, since normal cellular levels are far too low to be detected. A previous study of lung tumours (Iggo *et al.*, 1990) examined 47 cases and found that 60% of the tumours stained positively for p53. Our aim was to extend the study to 125 cases of primary lung tumours and also to see if any correlation could be found between p53 over-expression and survival. Should a link be found between p53 expression and survival in lung cancer, the staining procedure could prove to be a valuable diagnostic indicator and might assist in the selection of appropriate therapy.

In this study we have examined 125 primary lung cancers of the major histological types and stained them with a panel of antibodies for the presence of elevated levels of p53. The results have then been analysed with respect to patient survival and tested statistically for any correlation between p53 expression and survival.

### Materials and methods

### *Lung specimens*

One hundred and twenty-five tumours were received immediately after removal as lung resection specimens from the operating theatre. These comprised tumours from patients undergoing radical pulmonary resection for carcinoma of the lung in Oxford and for whom satisfactory clinical data could be obtained. They were collected between 1984 and 1988, with a mean follow-up period of 31.5 months; 49% of the patients had died by the end of the study (96% of whom were assumed to have died of their disease).

Histological classification and differentiation were assessed by light microscopy independently prior to immunocytochemical staining. Tumours were classified as small cell (five cases), squamous cell (78 cases) and adenocarcinoma (40 cases) according to the predominant cell type seen on light microscopy as described previously by the authors (Dunnill & Gatter, 1986).

Correspondence: K.C. Gatter, Nuffield Department of Pathology, John Radcliffe Hospital, Headington, Oxford OX3 9DU, UK.
Received 20 January 1992; and in revised form 10 April 1992.

All tissue was frozen in liquid nitrogen and stored at − 70°C until required. 8 μm cryostat sections were taken from each tumour and dried overnight to improve cellular morphology, prior to fixation in acetone for 10 min. Sections were either used immediately or stored at − 20°C.

The primary antibodies (see Table I) were applied to the dry tissue sections and incubated in a moist chamber for 30 min. The sections treated with polyclonal antibodies were then incubated with hybridoma supernatant from a mouse monoclonal antibody against rabbit immunoglobulins. Rabbit-anti-mouse immunoglobulins and then pre-formed anti-alkaline phosphatase (APAAP) complexes were then added to all sections, initially for 30 min each and then for a further 10 min to intensify the staining reaction. The sections were washed in Tris-buffered saline (TBS) between every stage.

Staining was achieved by the APAAP method (Cordell *et al.*, 1984), followed by counterstaining with haematoxylin. Control sections for each tumour had the primary antibodies omitted. Two authors (RM & IK) made separate microscopic examinations of all slides, noting the presence of nuclear positivity; this was done independently and results were compared afterwards. Positive staining of less than 10%, or very weak diffuse staining was considered negative.

Survival curves were plotted using the method of Kaplan and Meier (Kaplan & Meier, 1958) with statistical significance calculated using the log rank test (Peto *et al.*, 1977).

### Results

Significant positive staining was detected in 70 of the 125 tumour specimens examined (54%). In spite of the long storage of many of the specimens no deterioration in the quality of staining could be detected. The results for the different histological types are shown in Table II.

Positive staining was found to be a predominantly nuclear phenomenon (Figure 1), although some cells showed slight traces of reactivity within the cytoplasm. A few nuclei demonstrated unusual granular positivity, in an otherwise negative case; such positivity was disregarded.

The intensities of staining resulting from the different antibodies varied greatly but consistently. CM-1, PAb1801 and C19 all produced quite intense staining, whilst PAb421 and PAb240 were much weaker (Figure 2), with only a few cases showing strong, clear positivity. Positive staining was generally most intense around the periphery of each tumour island, although several cases showed very patchy positivity, in which scattered cells throughout the island were stained.

Survival graphs of all cases and squamous or adenocarcinomas separately showed parallel curves with no statistical difference between negative and positive cases (Figure 3). The survival curves were also plotted for individual antibodies. Again as above there were no statistically significant differences. (Figure 3).

**Figure 1** Detail of squamous cell carcinoma of the lung which showed strong homogenous expression of p53 protein expression with all 5 monoclonal antibodies used in this study. **a**, antibody PAb1801 and **b**, antibody C19 illustrate the range of staining intensity seen.

### Discussion

Mutations of the p53 gene were first implicated in tumorigenesis in 1985 when Jenkins *et al.* (Jenkins *et al.*, 1985) discovered that the transforming activity of p53 in the murine system was enhanced following mutation of its amino acid sequence. Subsequently it has been declared the most frequently mutated tumour suppressor gene in human tumours. A previous study of lung tumours (Iggo *et al.*, 1990)

**Table I** Antibodies used in this study

| *Antibody* | *Position of recognised epitope on P53 protein* | *Ref/source* |
|---|---|---|
| *Monoclonal:* | | |
| PAb1801 | between amino acids 32 and 79 | (Banks *et al.*, 1986) |
| PAb240 | between amino acids 156 and 335 | (Gannon *et al.*, 1990) |
| PAb421 | between amino acids 370 and 378 | (Harlow *et al.*, 1981) |
| *Polyclonal:* | | |
| CM-1 | both recognise a number of | David Lane |
| C19 | different sites on the protein | David Lane |

**Table II** Summary of results

| *Tumour type* | *No. of specimens* | *No. positive (%)* | *No. negative (%)* |
|---|---|---|---|
| SQC | 78 | 46 (59) | 32 (41) |
| ADC | 42 | 21 (50) | 21 (50) |
| SCC | 5 | 1 (20) | 4 (80) |

Figure 2 An adenocarcinoma of the lung which showed variable staining with the panel of antibodies ranging from strong homogeneous positivity with antibody PAb 1801 **a**, down to virtual negativity with antibody PAb 421 **b**.

examined 47 cases and found that 60% of the tumours stained positively for p53. Our aim was to extend the study to 125 cases of primary lung tumours and also to see if any correlation could be found between p53 over-expression and survival.

We found that 54% of the tissue samples stained positively for p53, indicating the presence of excessive levels of the protein; abnormal levels are the result of p53 stabilisation, which is believed only to occur with a mutated protein. This percentage confirms our earlier study (Iggo *et al.*, 1990) and compares favourably with the results from another smaller study on lung tumours (50% (Caamano *et al.*, 1991)) and with work on breast (45.5%) (Cattoretti *et al.*, 1988), colorectal (55%) (Van Den Berg *et al.*, 1989) and (42%) (Scott *et al.*, 1991) and ovarian cancer (50%) (Marks *et al.*, 1991).

However, it is important to recognise that there could be many tumours which are deficient in wild-type p53, yet do not register as positive in our study. The most likely abnormality in such cases is the loss of both alleles for p53 (Mowat *et al.*, 1985), so that neither wild-type nor mutant p53 is expressed. Alternatively a mutation could occur that produces a p53 molecule which is not recognized by any of the five antibodies in our panel. Yewdell *et al.* (1986) have also described the phenomenon of selective p53 phosphorylation and protein complexing in tumour cells, which may conceivably have significant effects upon epitope availability.

Our results showed that from a sample of 125 lung tumours, there was no statistically significant correlation

Figure 3 Survival curves for p53 protein expression for **a**, all lung tumours **b**, squamous cell carcinomas **c**, adenocarcinomas of the lung.

between the level of expression of mutant p53 proteins and patient survival, either for the entire range of tumours or for specific histological types. In a study of 51 non-small cell tumours (Chiba *et al.*, 1990) no significant association could be found with tumour stage, nodal status or histological type. Although no survival data were given these results support the findings of the present study. Correlations between p53 and survival have been reported on two occasions in breast cancer (Cattoretti *et al.*, 1988; Thompson *et al.*, 1990), although in the case of Cattoretti (Cattoretti *et al.*, 1988) it appears to be merely an association between mutant p53 and the expression of another protein, rather than intrinsically linking p53 to survival. Scott (Scott *et al.*, 1991) found a link in colorectal cancer between p53 expression and cell proliferation, indicating that a deficiency of wild-type p53 may prevent dividing cells from becoming quiescent. This would maintain an excessive number of cells in the dividing pool, conceivably enhancing the aggression of the tumour. However, despite such a finding, Scott (Scott *et al.*, 1991) was also unable to link p53 statistically with survival (negative Dukes test result). Furthermore, although they used only one antibody, Ostrowski *et al.* (Ostrowski *et al.*, 1991) couldn't show any correlation between labelling and survival in breast tumours.

The consistent lack of positive correlations suggests that although p53 is of considerable importance in the initiation of tumours in a wide variety of tissues, the nature of the particular oncogene involved initially is probably of little significance once a tumour has developed (although this may be proven wrong in future molecular studies on a variety of p53 mutations). We can conclude that in lung cancer the level of expression of p53 does not significantly affect the length of patient survival and is therefore at present of no use as a diagnostic indicator for this disease.

However, it is likely that once a tissue has become cancerous, the probability of survival is not decreased by overexpression of p53, since those tumours indicated as negative for p53 will presumably have a mutation in another gene, leading to enhanced cell division by other means. Thus once a tissue is cancerous, the particular oncogene involved initially will probably play little part in the prognosis. It should also be remembered that unlabelled tumours may also contain gross abnormalities in p53 expression since the causative cell could feasibly have possessed a double deletion of the p53 allele.

### References

BANKS, L., MATLASHEWSKI, G. & CRAWFORD, L. (1986). Isolation of human-p53-specific monoclonal antibodies and their use in the studies of human p53 expression. *Eur. J. Biochem.*, **159**, 529–534.

BARTEK, J., IGGO, R., GANNON, J. & LANE, D. (1990). Genetic and immunohistochemical analysis of mutant p53 in human breast cancer cell lines. *Oncogene*, **5**, 893–899.

CAAMANO, J., RUGGERI, B., MOMIKI, S., SICKLER, A., ZHANG, S. & KLEIN-SZANTO, A. (1991). Detection of p53 in primary lung tumors and nonsmall cell lung carcinoma cell lines. *Am. J. Pathol.*, **139**, 839–845.

CATTORETTI, G., RILKE, F., ANDREOLA, S., D'AMATO, L. & DELLA, D. (1988). P53 Expression in Breast Cancer. *Int. J. Cancer*, **41**, 178–183.

CHIBA, I., TAKAHASHI, T., NAU, M.M., D'AMICO, D., CURIEL, D.T., MITSUDOMI, T., BUCHHAGEN, D.L., CARBONE, D., PIANTADOSI, S. & KOGA, H. (1990). Mutations in the p53 gene are frequent in primary, resected non-small cell lung cancer. Lung Cancer Study Group. *Oncogene*, **5**, 1603–1610.

CORDELL, J.L., FALINI, B., ERBER, W.N., GHOSH, A.K., ABDULAZIZ, Z., MACDONALD, S., PULFORD, K.A.F., STEIN, H. & MASON, D.Y. (1984). Immunoenzymatic labelling of monoclonal antibodies using immune complexes of alkaline phosphatase and monclonal anti-alkaline phosphatase (APAAP complexes). *J. Histochem. Cytochem.*, **32**, 219–229.

DUNNILL, M.S. & GATTER, K.C. (1986). Cellular heterogeneity in lung cancer. *Histopathology*, **10**, 461–475.

GANNON, J.V., GREAVES, R., IGGO, R. & LANE, D.P. (1990). Activating mutations in p53 produce a common conformational effect. A monoclonal antibody specific for the mutant form. *Embo. J.*, **9**, 1595–1602.

HARLOW, E., CRAWFORD, L.V., PIM, D.C. & WILLIAMSON, N.M. (1981). Monoclonal antibodies specific for simian virus 40 tumour antigens. *J. Virol.*, **39**, 861–869.

HERSHOWITZ, I. (1987). Functional inactivation of genes by dominant negative mutations. *Nature*, **329**, 219–222.

IGGO, R., GATTER, K.C., BARTEK, J., LANE, D.P. & HARRIS, A.L. (1990). Increased expression of mutant forms of p53 oncogene in primary lung cancer. *Lancet*, **335**, 675–679.

JENKINS, J.R., RUDGE, K., CHUMAKOV, P. & CURRIE, G.A. (1985). The cellular oncogene p53 can be activated by mutagenesis. *Nature*, **317**, 816–818.

KAPLAN, E.L. & MEIER, P. (1958). Non parametric estimation from incomplete observations. *J. Am. Stat. Ass.*, **53**, 457–481.

LANE, D.P. & CRAWFORD, L.V. (1979). T antigen is bound to a host protein in SV40-transformed cells. *Nature*, **278**, 261–263.

MARKS, J.R., DAVIDOFF, A.M., KERNS, B.J., HUMPHREY, P.A., PENCE, J.C., DODGE, R.K., CLARKE-PEARSON, D.L., IGLEHART, J.D., BAST, R.C. & BERCHUCK, A. (1991). Overexpression and mutations of p53 in epithelial ovarian cancer. *Cancer Res.*, **51**, 2979–2984.

MERCER, W.E., AVIGNOLO, C. & BASERGA, R. (1984). Role of the p53 protein in cell proliferation as studied by microinjection of monoclonal antibodies. *Mol. Cell. Biol.*, **4**, 276–281.

MOWAT, M., CHENG, A., KIMURA, N., BERNSTEIN, A. & BENCHIMOL, S. (1985). Rearrangements of the cellular p53 gene in erythroleukaemic cells transformed by Friend virus. *Nature*, **314**, 633–636.

NIGRO, J.M., BAKER, S.J., PREISINGER, A.C., JESSUP, J.M., HOSTETTER, R., CLEARLY, K., BIGNER, S.H., DAVIDSON, N., BAYLIN, S., DEVILEE, P., GLOVER, T., COLLINS, F.S., WESTON, A., MODALI, R., HARRIS, C.C. & VOGELSTEIN, B. (1989). Mutations in the p53 gene occur in diverse human tumour types. *Nature*, **342**, 705–708.

O'ROUKE, R.W., MILLER, C.W., KATO, G.J., SIMON, K.J., CHEN, D.-L., DANG, C.V. & KOEFFER, H.P. (1990). A potential transcriptional activation element in the p53 protein. *Oncogene*, **5**, 1829–1832.

OSTROWSKI, J.L., SAWAN, A., HENRY, L., WRIGHT, C., HENRY, J.A., HENNESSY, C., LENNARD, T.J.W., ANGUS, B. & HORNE, C.H.W. (1991). p53 Expression in human breast cancer related to survival and prognostic factors: an immunohistochemical study. *J. Pathol.*, **164**, 75–81.

PETO, R., PIKE, M.C., ARMITAGE, P., BRESLOW, N.E., COX, D.R., HOWARD, S.V., MANTEL, N., McPHERSON, K., PETO, J. & SMITH, P.G. (1977). Design and analysis of randomised clinical trials requiring prolonged observation of each patient. *Br. J. Cancer*, **35**, 1–39.

PINHASI, K.O., MICHALOIVITZ, D., BEN, Z.A. & OREN, M. (1986). Specific interaction between the p53 cellular tumour antigen and major heat shock proteins. *Nature*, **320**, 182–184.

PURDIE, C.A., O'GRADY, J., PIRIS, J., WYLLIE, A.H. & BIRD, C.C. (1991). p53 expression in colorectal tumors. *Am. J. Pathol.*, **138**, 807–813.

SCOTT, N., SAGAR, P., STEWART, J., BLAIR, G.E., DIXON, M.F. & QUIRKE, P. (1991). p53 in colorectal cancer: clinicopathological correlation and prognostic significance. *Br. J. Cancer*, **63**, 317–319.

THOMPSON, A.M., STEEL, C.M., CHETTY, U., HAWKINS, R.A., MILLER, W.R., CARTER, D.C., FORREST, A.P. & EVANS, H.J. (1990). p53 gene mRNA expression and chromosome 17p allele loss in breast cancer. *Br. J. Cancer*, **61**, 74–78.

VAN DEN BERG, F.M., TIGGES, A.J., SCHIPPER, M.E., FC, D.H.J., KROES, W.G. & WALBOOMERS, J.M. (1989). Expression of the nuclear oncogene p53 in colon tumours. *J. Pathol.*, **157**, 193–199.

YEWDELL, J.W., GANNON, J.V. & LANE, D.P. (1986). Monoclonal antibody analysis of p53 expression in normal and transformed cells. *J. Virol.*, **59**, 444–452.